# Piecing Together the History of Protein Folds From a Fragmented Evolutionary Record

**DOI:** 10.1093/gbe/evaf148

**Published:** 2025-08-21

**Authors:** Claudia Alvarez-Carreño

**Affiliations:** Department of Structural and Molecular Biology, University College London, London, UK

**Keywords:** fold change, domain swapping, circular permutation, protein evolution, protein folding

## Abstract

Protein folds are structural units defined by the number, type, arrangement, and orientation of their core secondary structural elements. The universe of protein folds is highly interconnected. Local sequence similarities, referred to as sequence motifs, link structurally distinct folds. Sequence and structure motifs reveal deep evolutionary relationships that can help us understand the evolutionary mechanisms shaping protein structures over time. This work analyses structural divergence in folds that contain the β-hammerhead motif. Sequence and structure-based analyses reveal deep evolutionary relationships between 3-fold superfamilies: Beta Barrel (CATH superfamily 2.40.50.100); Distorted Sandwich (CATH superfamily 2.70.70.100); and Alpha-Beta Complex (CATH superfamily 3.90.1170.30). The patterns of fold divergence and motif degeneration are discussed in the context of fold evolution.

Significance This work follows the divergence folds that contain a β-hammerhead motif (βHM). The βHM is found in globally distinct folds and in diverse enzymes such as phosphoenolpyruvate-protein phosphotransferase, thymidine phosphorylase and nicotinate-nucleotide pyrophosphorylase. A detailed bioinformatics analysis reveals differing windows of sequence and structure similarity. The pattern of conservation suggests that βHMs are conserved structural features inherited from ancestral proteins rather than distinct and invariable units. Sequence- and structure-based analyses provide complementary insights into the origins of protein fold diversity.

## Introduction

Globular proteins exhibit compact folds formed by either sequential or nonsequential regions of the polypeptide chain. Folds are characterized by the number, nature and arrangement of core secondary structural elements (SSEs) and their spatial orientation (Chothia 1992; Orengo et al. 1997). Nature discovered protein folds more than 3.5 billion years ago.

The universe of protein structures is made of a relatively small number of folds (Koonin et al. 2002; Levitt 2009). Classification systems currently recognize up to seven thousand folds in experimentally determined protein structures [CATH superfamilies: 6573 (Sillitoe et al. 2021); SCOP families: 5936 (Andreeva et al. 2020); ECOD T-groups: 3950 (Schaeffer et al. 2024)]. TED [The Encyclopedia of Domains (Lau et al. 2024)], a classification of predicted structural data from the AlphaFold Protein Structure Database (Varadi et al. 2022), has identified an additional 7,000 new folds.

Advances in experimental structure determination and structure prediction methods have provided unprecedented access to protein structural data. Despite these advances, our understanding of protein fold evolution remains incomplete. Key questions persist, how do new protein folds emerge? How do protein structures change over evolutionary time?

### The Protein Universe is Connected

Multiple analyses of the sequence space show sequence similarities between folds that appear structurally distinct (Friedberg and Godzik 2005; Alva et al. 2015; Ferruz et al. 2020; Kolodny et al. 2021). Characteristically, sequence similarity across folds is not global but local (i.e. it does not span the entire fold) (Ferruz et al. 2020). Local regions of similarity across folds, called here *motifs*, can occur in the primary structure (sequence motifs) in the tertiary structure (structural motifs), or in both. Some of the most abundant protein folds share sequence and structural motifs (Kolodny et al. 2021), for example, flavodoxin-like and TIM barrel (Farías-Rico et al. 2014); Rossmann-like folds (Medvedev et al. 2021); cradle loop barrels (Alva et al. 2008); and OB-fold and SH3 (Alvarez-Carreño et al. 2021).

Motifs retain information about deep ancestral relationships and are central to models of fold emergence (Fetrow and Godzik 1998; Lupas et al. 2001; Alva et al. 2015; Kolodny et al. 2021). Some models of fold emergence propose that the first protein folds originated from small, nonfolding proteins with specific sequence motifs (antecedent motifs), which later evolved into structured proteins through duplication, combinatorial shuffling, fusion, and/or insertions (Lupas et al. 2001; Söding and Lupas 2003). In these models, sequence motifs antecede structural motifs.

Although most folds emerged near the dawn of life, several folds formed by single repetitive motifs appear to have evolved recently (Pereira and Lupas 2022). It appears that internal duplication is a common and ongoing mechanism of fold emergence.

### Shared Motifs as Byproducts of Protein Evolution

**Fig. 1.**
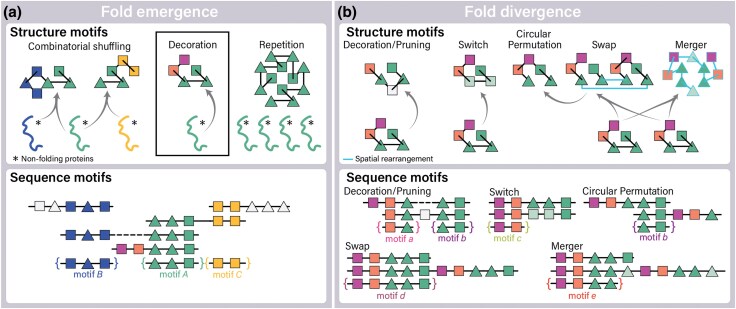
Mechanisms of fold emergence and evolution. Schematic representation of changes in the arrangement of SSEs. Squares represent β-strands; triangles represent helices. a) Model of fold emergence by combinatorial shuffling, decoration and repetition of antecedent motifs. b) Mechanisms of fold divergence: decoration and pruning of SSEs; switch; circular permutation; domain swapping; and merger. Circularly permuted, swapped and merged folds originate not from a single ancestral fold but from multiple ancestors. During fold divergence, structural motifs can degenerate (lighter colors). Lower panels: different mechanisms of fold emergence and evolution result in different sequence motifs. Left: motifs A, B and C are antecedent motifs. Right: Motifs a, b, d and e include part of antecedent motif A. Only motif d contains the complete antecedent motif A.

Motifs propagate through an additional process, fold divergence (Grishin 2001). Fold divergence begins with a protein that is already capable of folding (Fig. 1b). As proteins evolve, their folds can undergo substantial changes, which may include changes to core SSEs. During this process, sequence and structural motifs not only propagate but also degenerate (Alvarez-Carreño et al. 2022). Degeneracy of structural motifs consists of loss of structure similarity with or without preservation of sequence similarity. SSEs of a fold may be added or lost by decoration and pruning (Grishin 2001; Prakash and Bateman 2015); or interconverted (fold switching) (Chakravarty et al. 2023); or exchanged with SEEs of other folds (merger, swap, and circularly permutation) (Alvarez-Carreño et al. 2022).

Both fold emergence and fold divergence processes result in shared motifs, which poses a challenge for analysis. In the fold emergence scenario, a specific sequence motif gives rise to a distinct structural motif with defined boundaries (Fig. 1a). In contrast, fold divergence begins with an already folded ancestor and involves changes in both sequence and structure (Fig. 1b). In the scenario of fold divergence, structural motifs are regions of structural conservation between diverged folds. The location and extent of structural changes determine the boundaries of the shared motifs (Grishin 2001). Interpretations of the evolutionary history of motifs differs fundamentally between these two scenarios.

Here, I examine the evolution of proteins related to the β-hammerhead motif (βHM). This motif is shared among folds with distinct topologies (Alva et al. 2015; Schaeffer et al. 2016) (Table 1) including mainly β structures (e.g. cytochrome C, C-terminal domain; and the *N*-terminal domain of phosphoenolpyruvate–protein phosphotransferase) and α+β structures (e.g. thymidine phosphorylase, C-terminal domain; and nicotinate-nucleotide pyrophosphorylase [carboxylating], N-terminal domain). The βHM has been interpreted as an antecedent motif from which all these diverse folds originated (Alva et al. 2015).

**Table 1:** Examples of domains that contain a βHM

UniProt ID	Protein name	Source	TED	Residue range	CATH superfamily
A0A2S0L278	DNA-directed RNA polymerase subunit beta'	*Mogibacterium diversum*	TED06	954 to 1012	2.40.50.100
B5YFV7	DNA-directed RNA polymerase subunit beta'	*Thermodesulfovibrio yellowstonii* (strain ATCC 51303/DSM 11347/YP87)	TED05	1015 to 1108	2.40.50.100
A2BPU4	cytochrome f	*Prochlorococcus marinus* (strain AS9601)	TED03	207 to 260	2.40.50.100
A0A356FX18	phosphoenolpyruvate–protein phosphotransferase	*Alphaproteobacteria bacterium*	TED01	7 to 157	2.70.70.10
A0A7X6YQS1	thymidine phosphorylase	*Clostridiaceae bacterium*	TED02	121 to 208	3.90.1170.30

**Fig. 2.**
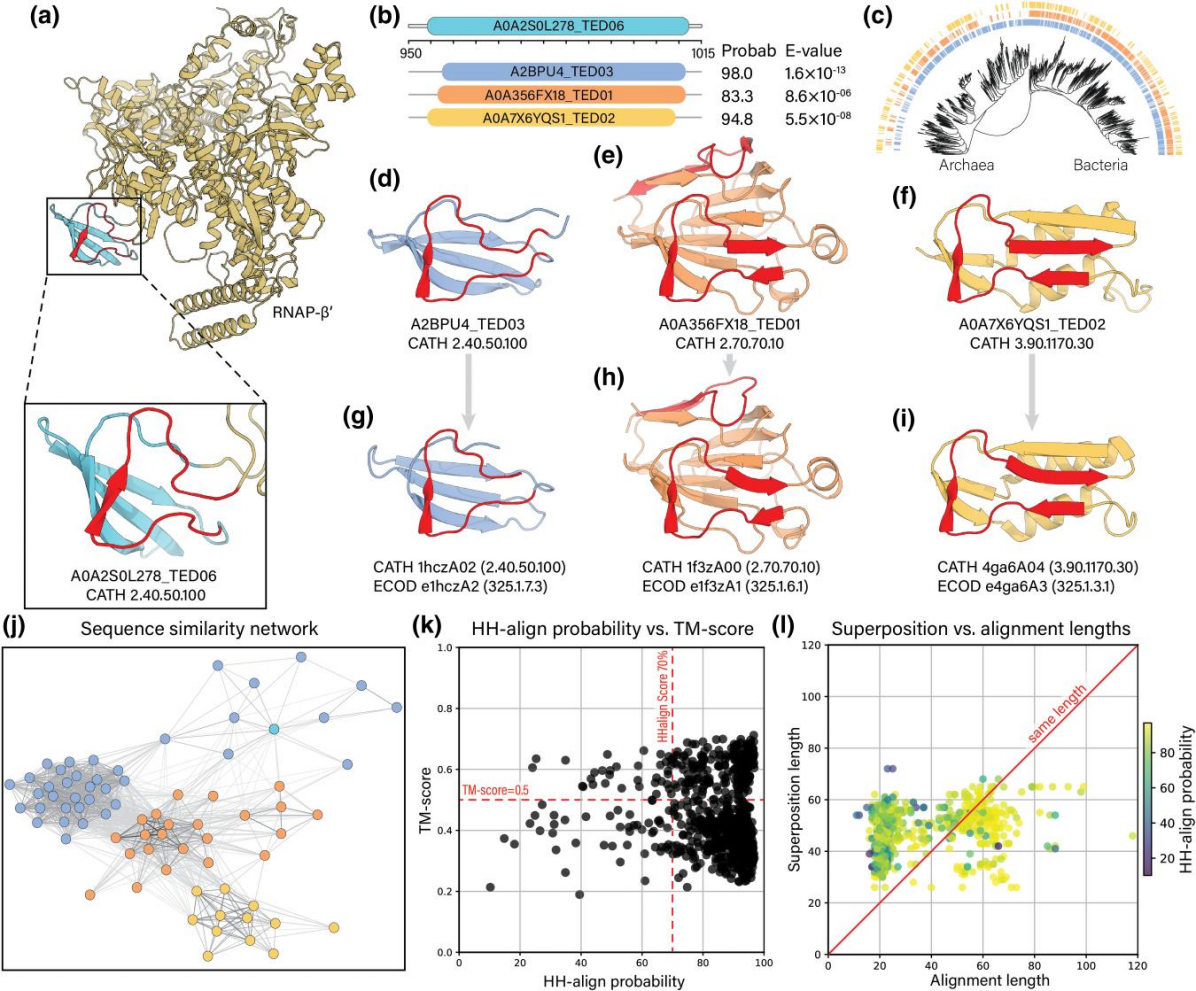
Sequence and structure similarities between folds with a βHM. a) Structure of the β″ subunit of the DNA-directed RNA polymerase from Mogibacterium diversum. Inset: bacteria-specific insertion β′-bD6 corresponding to domain A0A2S0L278_TED06. b) Results of a pairwise HMM-HMM comparisons between the query domain A0A2S0L278_TED06 and three target domains in TED: A2BPU4_TED03; A0A356FX18_TED01; and A0A7X6YQS1_TED02. c) Phylogenetic distribution of CATH superfamilies 2.40.50.100 (inner strip); 2.70.70.10 (middle strip); and 3.90.1170.30 (external strip) mapped into a tree of bacteria and archaea modified from (Moody et al. 2022). d) Structure of domain A2BPU4_TED03 in cytochrome f from Prochlorococcus marinus. e) Structure of domain A0A356FX18_TED01 in phosphoenolpyruvate–protein phosphotransferase from an Alphaproteobacteria bacterium. f) Structure of domain A0A7X6YQS1_TED02 in thymidine phosphorylase from a Clostridiaceae bacterium. g) Structure of 1hczA02 (CATH superfamily 2.40.50.100). h) Structure of 1f3zA00 (CATH superfamily 2.70.70.10). i) Structure of 4ga6A04 (CATH superfamily 3.90.1170.30). j) Network of sequence similarities between domains in superfamilies 2.40.50.100, 2.70.70.100, and 3.90.1170.30. Colors follow those used in (b). The network shows connections between HMMs that align with an *E*-value < 1 × 10^−7^ calculated by HH-align. k) TM-score versus HH-align probability of pairwise comparisons across fold superfamilies. Domains within 2.40.50.100 were compared with domains within superfamilies 2.70.70.100 and 3.90.1170.30. l) Superposition versus alignment length of similarities across fold superfamilies.

An initial search for folds containing the βHM was made using a hammerhead/barrel-sandwich hybrid (HABAS) fold insertion observed in the βʹ subunit of bacterial DNA-directed RNA polymerase (RNAP-βʹ). The HABAS fold is a mainly β structure that contains a βHM (Fig. 2a). In different bacterial lineages, HABAS is inserted at different positions in RNAP-βʹ. These HABAS insertions form a variety of structures (Fig. S1) through the exchange of SSEs (Chlenov et al. 2005; Alvarez-Carreño et al. 2024; Qayyum et al. 2024).

The present work focuses on divergence of proteins containing βHM from their ancestral folds while retaining regions of sequence similarity that extend beyond βHM. Through combined sequence and structure analyses, motif conservation and degeneration are detected at multiple levels, including subtle adjustments in individual SSEs, alterations in the interactions between these SSEs, and significant changes to hydrogen bond networks within the fold.

## Results

### Sequence Similarities Across Folds

The βHM is identified here in three distinct folds. To find these folds, a multiple sequence alignment (MSA) of the HABAS fold in RNAP-β″ was searched against a custom-made database of hidden Markov models (HMMs) of domains in TED (Lau et al. 2024) mapped to CATH (Sillitoe et al. 2021). High-scoring domains (*E*-value < 1 × 10^−5^; aligned length > 15 columns) were retrieved for analysis. The sequence similarity search retrieves domains within CATH superfamilies 2.40.50.100; 2.70.70.100 “Glucose Permease domain IIA”; and 3.90.1170.30 “Pyrimidine nucleoside phosphorylase-like, C-terminal domain”. Folds within superfamily 2.40.50.100 are structurally different from folds in superfamilies 2.70.70.100 and 3.90.1170.30 (Fig. 2d to i). Folds within these three superfamilies have homologs in bacteria and archaea (Fig. 2c), as well as homologs with experimentally determined three-dimensional structures (Fig. 2g to i).

To map sequence connections across superfamilies 2.40.50.100, 2.70.70.10, and 3.90.1170.30, HMMs were aligned to one another in an all-against-all fashion (see Methods). The cluster map of sequence similarities at an *E*-value of 1 × 10^−7^ (Fig. 2j) reveals extensive sequence similarity connections across domains in these three distinct superfamilies.

### Pairwise Sequence and Structure Comparisons Show Discrepancies Between Sequence and Structure Conservation

To understand whether regions of sequence similarity across folds match regions of structure similarity, 2.40.50.100 domains were superposed to 2.70.70.10, and 3.90.1170.30 domain structures using TM-align (Zhang and Skolnick 2005). For each pairwise comparison, the TM-score was plotted against the HH-align probability (Fig. 2k). Most pairs exhibit sequence similarities (HH-align probabilities above 70), however, the majority are structurally dissimilar (TM-scores below 0.5). This analysis also identified a group of pairs with high HH-align probabilities where the similarity region is longer in sequence than in structure (Fig. 2l). Three examples of sequence similarity with structure divergence (Fig. 2d, e and Table 1) were selected for detailed analysis. For description, helices are labeled as α, strands as β and loops as λ.

### Decoration

**Fig. 3.**
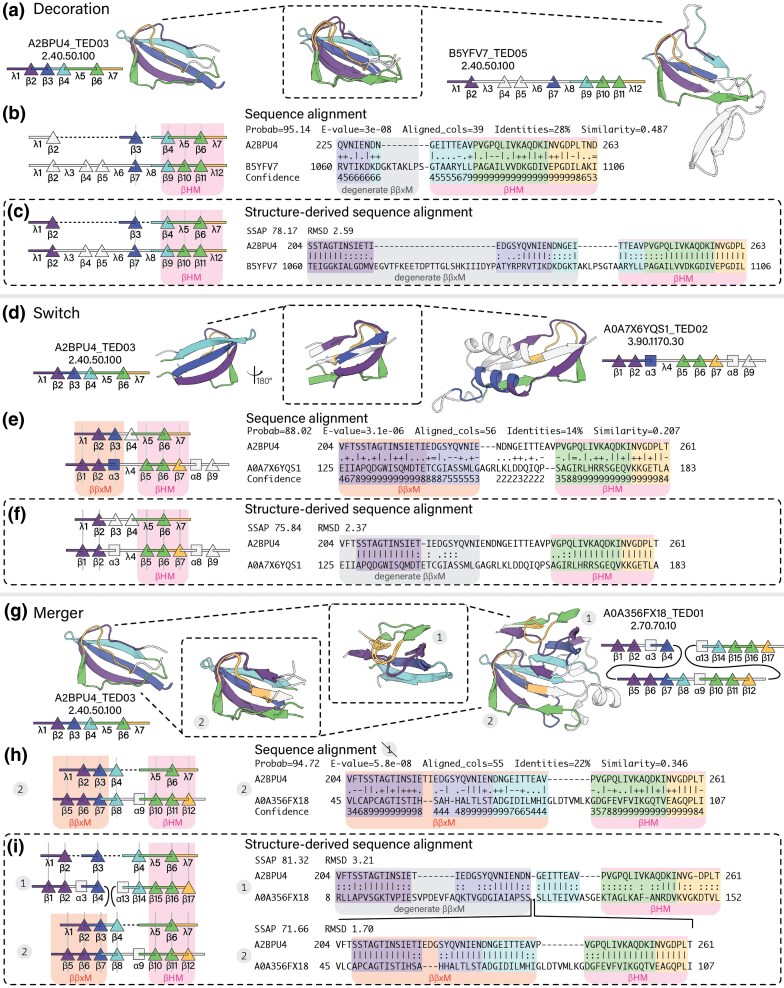
Cross-fold sequence similarities mapped onto structure. Pairwise similarities inferred from HMM-HMM comparisons or TM-align superpositions are indicated equivalent colors. a) Secondary and tertiary structure representations of A2BPU4_TED03 and B5YFV7_TED05. b) Primary and secondary structure representation of HH-align pairwise alignments between A2BPU4_TED03 and B5YFV7_TED05. c) Primary and secondary structure representation of the superposition-derived sequence alignments between A2BPU4_TED03 and B5YFV7_TED05. d) Secondary and tertiary structure representations of A2BPU4_TED03 and A0A7X6YQS1_TED02. e) Primary and secondary structure representation of HH-align pairwise alignments between A2BPU4_TED03 and A0A7X6YQS1_TED02. f) Primary and secondary structure representation of the superposition-derived sequence alignments between A2BPU4_TED03 and A0A7X6YQS1_TED02. g) Secondary and tertiary structure representations of A2BPU4_TED03 and A0A356FX18_TED01. h) Primary and secondary structure representation of HH-align pairwise alignments between A2BPU4_TED03 and A0A356FX18_TED01. i) Primary and secondary structure representation of the superposition-derived sequence alignments between A2BPU4_TED03 and A0A356FX18_TED01.

A2BPU4_TED03 and B5YFV7_TED05 are assigned to the same CATH superfamily (2.40.50.100) and have similar SSE arrangements. The main difference between these two domains is an insertion in B5YFV7_TED05 ([λ3β4β5]_B5YFV7_TED01_). Due to this long insertion, the only continuous region of sequence and structure similarity between A2BPU4_TED03 and B5YFV7_TED05 is βHM (Fig. 3b, c).

### Fold Switch

TED domain A2BPU4_TED03 (CATH 2.40.50.100) shows sequence similarity to A0A7X6YQS_TED02 (CATH 3.90.1170.30) (Fig. 3d to f), providing evidence for the hypothesis of a pathway of divergence between all-β and α+β βHM-containing folds (Schaeffer et al. 2016).

The *N*-terminal region of A2BPU4_TED03 ([λ1β2β3]_A2BPU4_TED03_) aligns to the *N*-terminal region of A0A7X6YQS_TED02 ([β1β2α3]_A0A7X6YQS_TED02_). This region of sequence similarity is called here ββx motif (ββxM). Sequence similarity between strand β3_A2BPU4_TED03_ and helix α3_A0A7X6YQS_TED02_ suggests that these SSEs may have interconverted in evolution. Interestingly, the MSA of the region that corresponds to helix α3_A0A7X6YQS_TED02_ shows a conserved β-strand propensity (Fig. S2), which adds support to the hypothesis that strand β3_A2BPU4_TED03_ and helix α3_A0A7X6YQS_TED02_ may have interconverted.

Between A2BPU4_TED03 and A0A7X6YQS_TED02, only βHM is preserved in both sequence and structure, but ββxM is degenerate in structure (Fig. 3f).

### Merger

A2BPU4_TED03 (2.40.50.100) has sequence and structure similarity to A0A356FX18_TED01 (2.70.70.100) (Fig. 3g to i). In sequence, ββxM ([λ1β2β3β4]_A2BPU4_TED04_ and [β5β6β7β8]_A0A356FX18_TED01_), and βHM ([λ5β6λ7]_A2BPU4_TED04_ and [β10β11β12]_A0A356FX18_TED01_) are detected once.

In structure, ββxM and βHM are detected twice in A0A356FX18_TED01. ββxM corresponds to [β1β2β4]_A0A356FX18_TED01_ and [β5β6β7]_A0A356FX18_TED01_; and βHM corresponds to [β10β11β12]_A0A356FX18_TED01_ and [β15β16β17]_A0A356FX18_TED01_. Overall, A2BPU4_TED03 maps to A0A356FX18_TED01 in two distinct regions: [β5β6β7β8α9β10β11β12]_A0A356FX18_TED01_ and [β1β2α3β4…β14β15β16β17]_A0A356FX18_TED01_. The pattern of structure similarity suggests that the fold in A0A356FX18_TED01 is a merger of two homologs of A2BPU4_TED03, one within the other (Fig. 3c).

### The βHM

Across the three cross-fold pairwise comparisons described above, the only motif that is consistently preserved both in sequence and structure is βHM. However, in all cases there are similarities either in sequence or structure that extend beyond this motif and include ββxM. All SSEs of A2BPU4_TED03 superpose to B5YFV7_TED05 (Fig. 3a); most SSEs in A2BPU4_TED03 align in sequence to A0A356FX18_TED01 (Fig. 3b); and all SSEs of A2BPU4_TED03 superpose to A0A356FX18_TED01 (Fig. 3c). The βHM appears to be a result of structural conservation; ββxM appears to be a degenerate motif.

## Discussion

Sequence and structure reflect different aspects of the evolutionary history of folds. Similarities between βHM-containing proteins, analysed here, can be explained by large insertions/deletions, changes in SSEs, and topological changes that result in degeneracy of structural motifs. These changes suggest that common divergent processes can allow a descendant fold to differ from its ancestral fold. The emerging fold may be exapted for a new function and can subsequently undergo selection for function or structural stability (Jayaraman et al. 2022).

Reconstruction of the evolutionary trajectories of folds requires a precise delineation of the boundaries of homologous relationships. Motifs are defined as distinct entities based on the results of comparative methods. For folds formed by nonrepeating units, motifs are identified by comparing pairs or groups of folds. The selection of folds for comparison, the definition of fold itself, and the sensitivity of the comparative methods affect the size and boundaries of the identified motifs (Nepomnyachiy et al. 2017). When folds that have diverged are compared, sequence and structural motifs show complex overlapping or discontinuous patterns (Alva et al. 2009; Nepomnyachiy et al. 2017), and their boundaries may exclude regions that are ancestrally related (Fig. 1b). Shared motifs, therefore, are not inherently distinct evolutionary units but instead represent distinctly conserved features within larger folds (Alvarez-Carreño et al. 2022).

As argued here, not all motifs originate during fold emergence; some (possibly most) may arise during fold divergence. Current maps of fold connections (Alva et al. 2010; Nepomnyachiy et al. 2014; Ferruz et al. 2020) do not differentiate between relationships established during fold emergence and those occurring during later fold evolution. Some of these deep relationships between protein folds can be inferred by combined sequence-structure methodologies such as the ones described here. Additionally, unexpected new folds have been discovered through experimental determination of protein constructs that have been designed to test the trajectories of fold evolution (Afanasieva et al. 2019; Yagi and Tagami 2024). These folds that lack an equivalent in the extant repertoire of proteins may represent ghost intermediates in protein fold evolution (Yagi and Tagami 2024).

βHM allows an exploration of the complex relationships between three extant protein fold superfamilies. Recent advances in protein structure prediction and design provide new approaches to understand protein evolution in general. By combining sequence- and structure-based phylogenetics, large-scale analysis of the universe of protein sequence and structures could uncover how the vast majority of folds emerged at the dawn of life.

## Methods

### Initial Sequence Similarity Search

RNAP-β sequences from 300 bacteria species were aligned with MAFFT (Katoh and Standley 2013). To build the sequence alignment (MSA) of the HABAS domain, the MSA of RNAP-β sequences was trimmed to the fold boundaries indicated in Table 1. The MSA was searched against a custom-made HMM database of TED domains (Lau et al. 2024) assigned to CATH superfamilies 2.40.50.100, 2.70.70.100, and 3.90.1170.30 with HH-search (Steinegger et al. 2019), and against CATH_S40 database with HHpred in the MPI web server (Gabler et al. 2020).

### Analysis of Cross-fold Similarities

HMMs in TED assigned to CATH superfamilies 2.40.50.100, 2.70.70.100, and 3.90.1170.30 were compared all-against-all with hhalign from HH-suite (Steinegger et al. 2019). Pairwise similarities across 73 HMMs were clustered using CLANS (Frickey and Lupas 2004) based on HH-align *E*-value up to 1 × 10^−7^.

Pairwise structure superpositions of TED domains (Lau et al. 2024 ) were computed locally with TM-align (Zhang and Skolnick 2005).

## Supplementary Material

evaf148_Supplementary_Data

## Data Availability

Raw outputs for the pairwise sequence and structure comparisons can be accessed in figshare (https://doi.org/10.6084/m9.figshare.28071164).
