# Supplementary material for: Piecing Together the History of Protein Folds From a Fragmented Evolutionary Record
Source: Genome Biol Evol. 2025 Aug 21;17(8):evaf148. doi: 10.1093/gbe/evaf148 (PMC12369578; doi:10.1093/gbe/evaf148)
Supplement: evaf148_Supplementary_Data [file evaf148_supplementary_data.pdf]

Supplementary Information

Piecing together the history of protein folds from a fragmented evolutionary record

Claudia Alvarez-Carreño[1]

[1]Department of Structural and Molecular Biology, University College London, London, United Kingdom

Claudia Alvarez-Carreño

Email: c.carreno@ucl.ac.uk

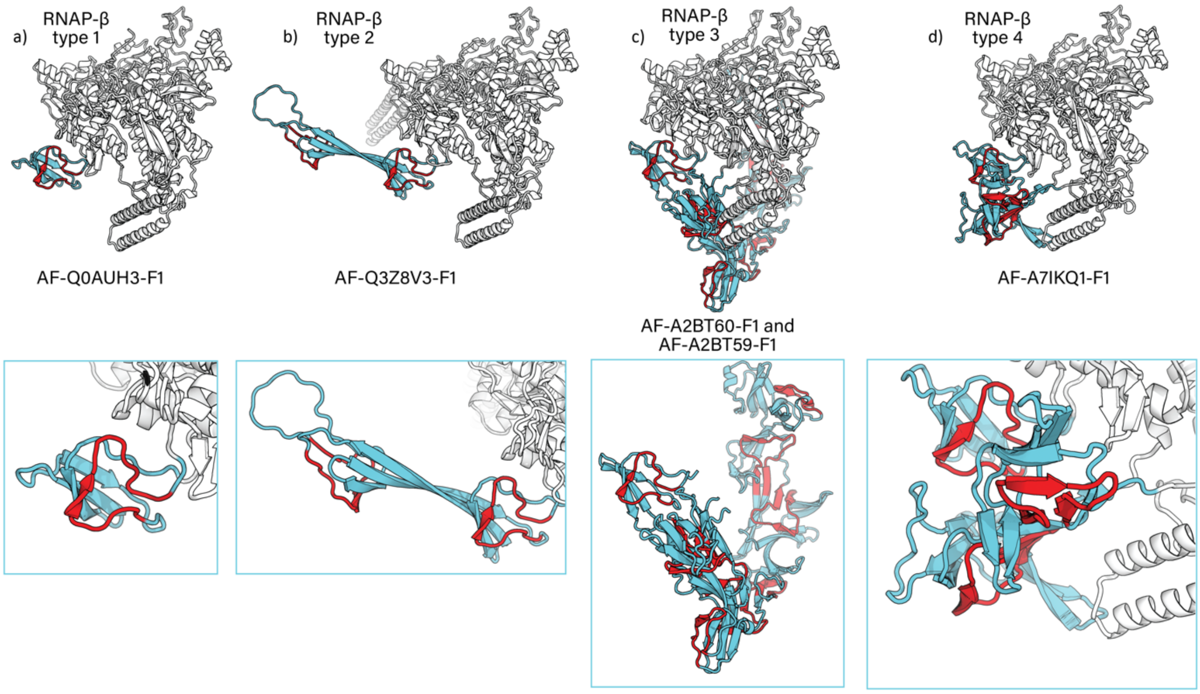

Supplementary Figure 1. Diversity of hammerhead/barrel-sandwich hybrid (HABAS) insertions in the β' subunit of bacterial DNA-directed RNA polymerases. a) Type 1 RNAP-β' from *Syntrophomonas wolfei* subsp. wolfei (AlphaFold DB: AF-Q0AUH3-F1), (b) type 2 RNAP-β' from *Dehalococcoides mccartyi* (AlphaFold DB: AF-Q3Z8V3-F1), (c) type 3 RNAP-β' from *Prochlorococcus marinus*. N-terminal fragment (AlphaFold DB: AF-A2BT60-F1) and C-terminal fragment (AlphaFold DB: AF-A2BT59 -F1). (d) type 4 RNAP-β' from *Xanthobacter autotrophicus* (AlphaFold DB: AF-A7IKQ1-F1). Adapted from (Alvarez-Carreño, et al. 2024).

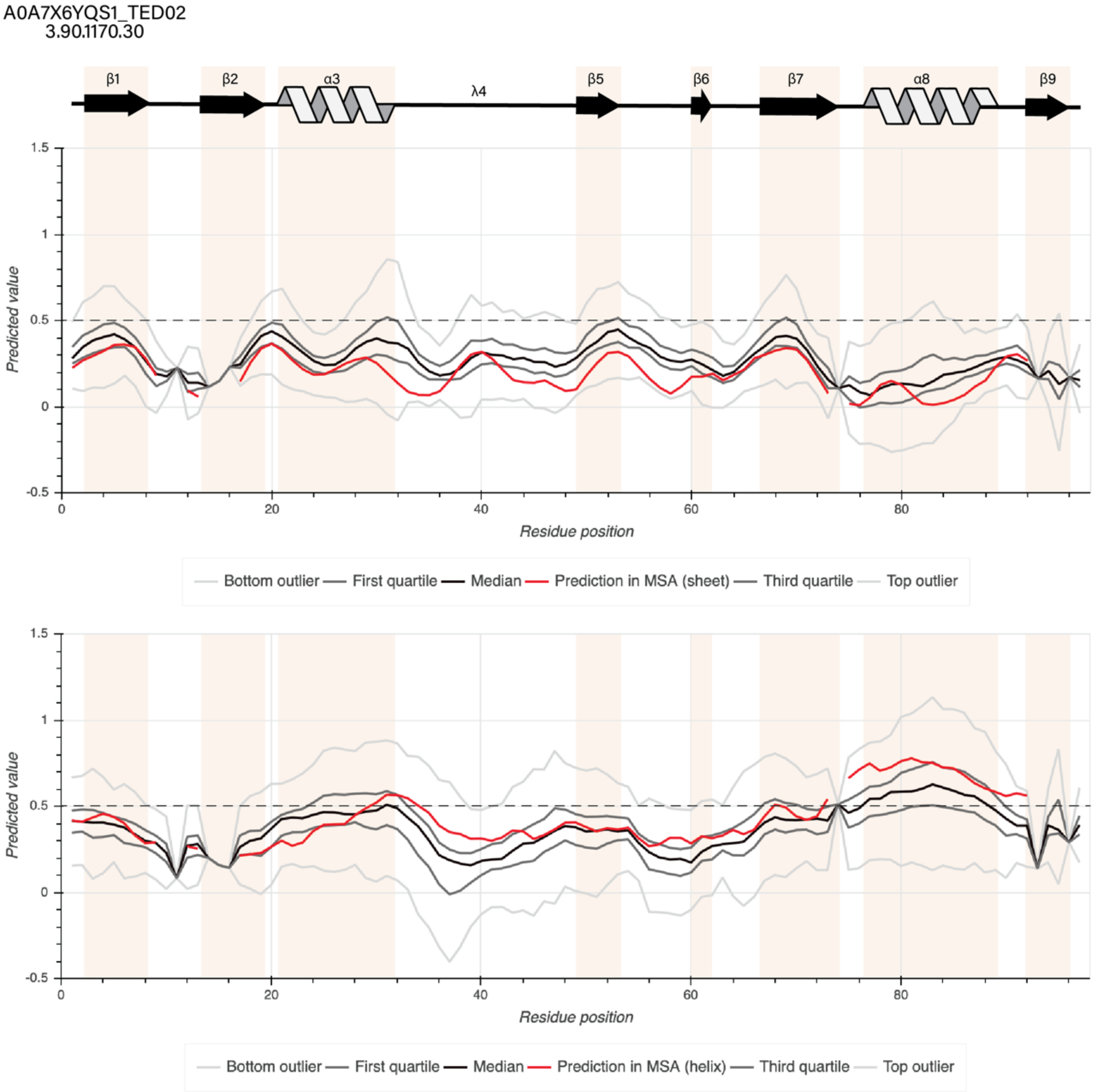

Supplementary Figure 2. Multiple sequence alignment derived distributions of predicted strand and helix propensities of domain A0A7X6YQS1_TED02. The distributions are based on an analysis of the multiple sequence alignment (MSA) of A0A7X6YQS1_TED02 homologs, showing the 'evolutionary allowed' range for this prediction. The red line corresponds to the single protein prediction for A0A7X6YQS1_TED02, the other lines show simple statistical parameters calculated from the values per MSA column. Predictions were calculated using DynaMine webserver (Cilia, et al. 2014).

References

Alvarez-Carreño C, Huynh AT, Petrov AS, Orengo C, Williams LD 2024. BEAN and HABAS: Polyphyletic insertions in the DNA-directed RNA polymerase. Protein Science 33: e5194. doi: 10.1002/pro.5194

Cilia E, Pancsa R, Tompa P, Lenaerts T, Vranken WF 2014. The DynaMine webserver: predicting protein dynamics from sequence. Nucleic Acids Research 42: W264-270. doi: 10.1093/nar/gku270
